# Caffeine Decreases Topiramate Levels in Zebrafish Larvae in a Pentylenetetrazol-Induced Seizure Model

**DOI:** 10.3390/ijms25063309

**Published:** 2024-03-14

**Authors:** Adrian Bartoszek, Agata Sumara, Anna Kozub-Pędrak, Alicja Trzpil, Anna Stachniuk, Emilia Fornal

**Affiliations:** Department of Bioanalytics, Medical University of Lublin, Ul. Jaczewskiego 8b, 20-090 Lublin, Poland

**Keywords:** caffeine, topiramate, zebrafish, zebrafish larvae, epilepsy, seizure, PTZ-induced seizure

## Abstract

Epilepsy ranks as the second-most prevalent neurological disease, and is characterized by seizures resulting in neurobiological and behavioral impairment. Naturally occurring in coffee beans or tea leaves, the alkaloid caffeine (CAF) is the most prevalent global stimulant. Caffeine has been observed to influence epileptic seizures and the efficacy of antiepileptic medications, with a notable impact on topiramate (TPM). This study aimed to explore the influence of CAF on TPM’s anticonvulsant effects in zebrafish larvae within a PTZ-induced seizure model, concurrently determining TPM concentrations through a sophisticated analytical approach based on ultrahigh-performance liquid chromatography and subsequent mass spectrometric detection. Zebrafish larvae four days post-fertilization were incubated for 18 h with varying doses of TPM or combinations of CAF + TPM, and locomotor activity was then assessed. Seizures were induced by introducing a PTZ solution to achieve a final concentration of 20 mM. Utilizing liquid chromatography–mass spectrometry (LC–MS/MS), TPM levels in the larvae were quantified. CAF co-administration (especially in higher doses) with TPM caused a decrease in the average locomotor activity in the larvae compared to TPM alone. Moreover, CAF decreased TPM levels in the larvae at all investigated doses. In conclusion, these findings offer a novel perspective on the interplay between CAF and TPM, shedding light on previously unexplored facets. The potential impact of CAF consumption in assisting with epileptic seizure control, unless proven otherwise, suggests a noteworthy consideration for future research and clinical practices.

## 1. Introduction

Epilepsy ranks as the second-most prevalent neurological disease. The first conceptualized definition of disease was made in 2005. It is defined by a persistent tendency to experience spontaneous epileptic seizures, leading to various neurobiological, cognitive, and psychosocial consequences [1]. In approximately 60% of cases, the cause of disease is unidentified. The highest incidence of epilepsy is observed in both young children and the elderly [2]. Patients typically experience physical consequences such as bruises and fractures following seizure episodes, as well as psychological challenges, including anxiety and depression [3]. Regrettably, although more than 30 antiepileptic drugs exist, a definitive cure for the condition has not been found, with only symptomatic treatments available [4]. Approximately one third of individuals with epilepsy do not respond to currently available medications [5]. To address this unmet need in clinical practice, there is a demand for novel animal models suitable for high-throughput screening of potential drug candidates. In recent years, the zebrafish (*Danio rerio*) has gained broad recognition within the scientific community as a valuable model for such endeavors due to its rapid development, high fecundity and small proportions [6]. It was presented that the dynamics of seizures in zebrafish and humans are remarkably similar, which enhances the transferability of results from the animal to humans [7].

Epilepsy has become a subject of interest for numerous research groups. Various substances, including those of plant origin, are being tested. One of them is 6-gingerol (6-GIN) isolated from *Zingiber officinale* by Gawel et al. [8]. The study employed a zebrafish larvae pentylenetetrazole (PTZ) seizure assay, demonstrating the anticonvulsant effect of *Zingiber officinale* rhizoma methanolic extract. Interestingly, the study contrasts with a previous one, possibly due to differences in extract composition and environmental factors. Therefore, the need for further exploration of potential plant-based phytotherapeutic interactions with other neurotransmitter systems and involvement in different seizure/epilepsy models is emphasized [8]. It is frequently noted that phytochemical compounds engage in interactions with the physiological systems of the human body, and they engage in interactions with bioactive compounds present in dietary constituents like CAF [9].

The most commonly consumed psychoactive substance globally is CAF, a member of the purine alkaloid group [10]. CAF naturally occurs in plants like coffee beans and tea leaves [10]. The average person consumes about 300 mg per day [10] of CAF from sources like tea, coffee, and soft drinks, which already constitutes a pharmacologically active dose [10]. Additionally, numerous food and beverage products contain added CAF, which can affect both epileptic seizures and the efficacy of anticonvulsant drugs, further complicating seizure management [11].

One of the medications used in epilepsy therapy is topiramate (TPM). TPM is a second-generation anticonvulsant drug whose primary use is in the treatment of epilepsy and migraines [12]. As a pharmaceutical agent, it is approved for use both as monotherapy and as adjunctive therapy. When administered alone, it exhibits linear pharmacokinetics (PK). However, various factors are known to influence its clearance, including age, concurrent medication use, and renal function [12].

Clinical data regarding the connection between CAF and epileptic seizures are limited. Most existing knowledge is derived from preclinical studies in animals, a handful of clinical trials, and a small number of case studies [13,14]. Present data underscore the intricate relationships between epileptic seizures, CAF, and antiepileptic drugs. As a result, clear clinical guidelines for the use of CAF in epileptic or at-risk individuals are difficult to establish. Interestingly, preclinical research suggests that CAF can increase susceptibility to epileptic seizures. In certain cases, CAF intake over long periods may offer protection against seizures. In previous studies, CAF diminished the effectiveness of numerous antiepileptic drugs, with TPM being most significantly affected. The relationships between CAF, epileptic seizures, and antiepileptic drugs continue to be complex and are not yet adequately understood [13,14].

As of now, as far as we know, a study comprehensively assessing the influence of CAF on the anticonvulsant action of TPM while quantifying the TPM concentrations in zebrafish larvae in a PTZ-induced seizure model does not exist.

The aim of this study was to investigate the effect of CAF on the anticonvulsant action of TPM in zebrafish larvae in a PTZ-induced seizure model. Additionally, we aimed to determine whether CAF influences TPM concentrations using an analytical method based on ultrahigh-performance liquid chromatography with subsequent mass spectrometric detection.

## 2. Results

### 2.1. The Influence of Caffeine and Topiramate Combinations on Larval Locomotor Activity

**Figure 1:**
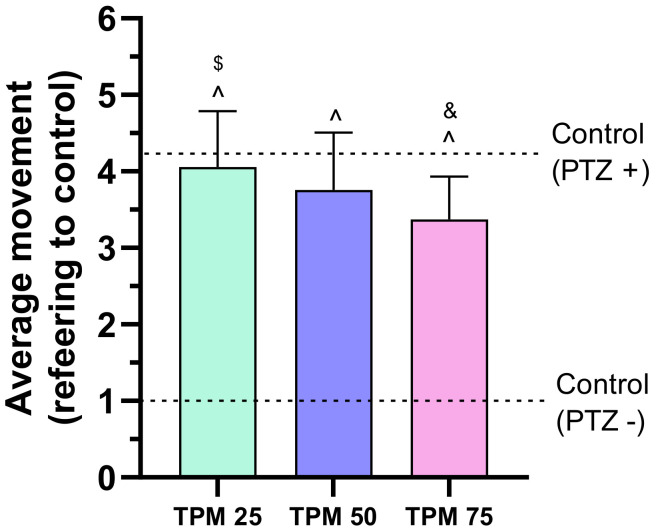
Effects of topiramate (TPM) on average larval locomotor movement in a PTZ-induced seizure model; TPM concentrations applied: 25, 50, 75 µM; ^ *p* < 0.05 compared to control (PTZ−), & *p* < 0.05 compared to control (PTZ+), $ *p* < 0.05 compared to TPM 75 µM group.

First, zebrafish larvae were treated with TPM (25 to 75 μM) and the average movement was assessed in a PTZ-induced seizure model (Figure 1). TPM in doses of 25 μM and 50 μM was not able to defend the larvae against PTZ. A 75 μM TPM dose suppressed movement significantly relative to the PTZ+ group, but did not lower it to the levels of the control PTZ− group.

**Figure 2:**
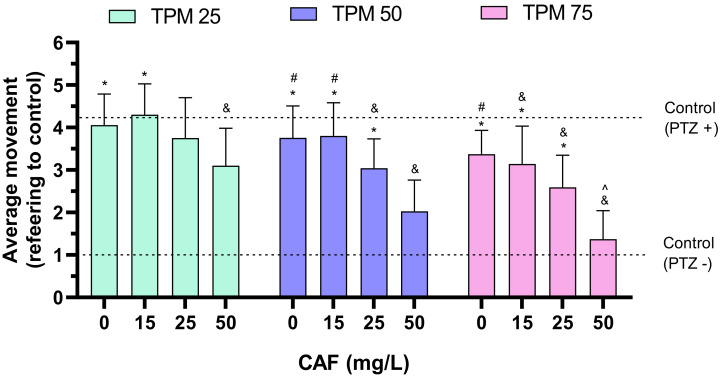
Effects of caffeine (CAF) and topiramate (TPM) combinations on average larval locomotor movement in a PTZ-induced seizure model; TPM concentrations applied: 25, 50, 75 µM; CAF concentrations applied: 15, 25, 50 mg/L; * *p* < 0.05, compared to CAF 50 mg/L group; # *p* < 0.05 compared to CAF 25 mg/L group, ^ *p* < 0.05—the only group not significantly different from control (PTZ−), & *p* < 0.05 compared to control (PTZ+).

Subsequently, the influence of CAF on TPM was assessed (Figure 2). Adding 50 mg/L CAF to 25 μM TPM significantly suppressed movement compared to larvae treated with 0 or 15 mg/L CAF. Only 50 mg/L CAF protected larvae against PTZ.

Adding 50 mg/L CAF to 50 μM TPM significantly reduced movement compared to groups administrated 0, 15, or 25 mg/L CAF. Also, treating the animals with 25 mg/L CAF reduced activity relative to the 0 and 15 mg/L CAF groups while treating with 50 μM TPM. Both 25 and 50 mg/L CAF protected larvae against PTZ.

Again, in the 75 μM TPM group, 50 mg/L CAF significantly reduced movement from lower CAF doses, while 15, 25 and 50 mg/L CAF protected larvae again PTZ.

CAF at 50 mg/L significantly suppressed zebrafish activity in co-administration with all investigated TPM dilutions compared to the PTZ+ group, but only the combination of 50 mg/L CAF with 75 μM TPM was able to lower it to the level of the PTZ− group.

### 2.2. Topiramate Quantification

**Figure 3:**
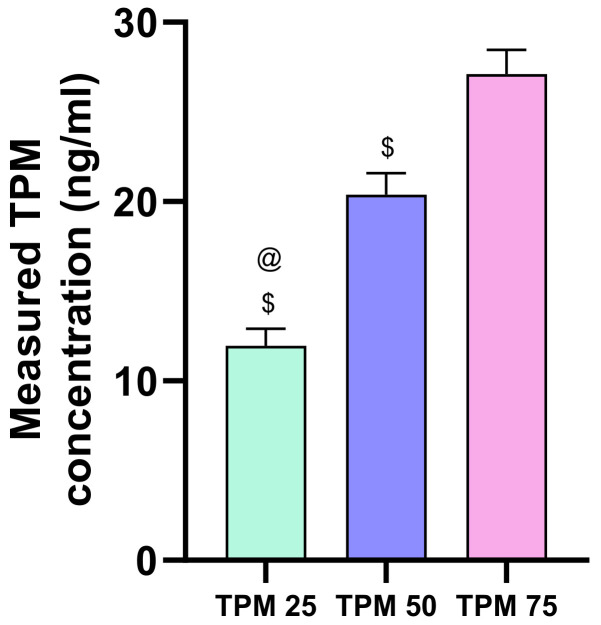
Contents of TPM for the larvae treated with TPM in PTZ-induced seizure model; TPM concentrations applied: 25, 50, 75 µm, @ *p* < 0.05 compared to TPM 50 µM group, $ *p* < 0.05 compared to TPM 75 µM group.

First, we determined the quantity of TPM accumulated in the zebrafish larvae in the dosages investigated above in larvae treated only with this compound. TPM levels differed significantly among all doses observed (Figure 3).

**Figure 4:**
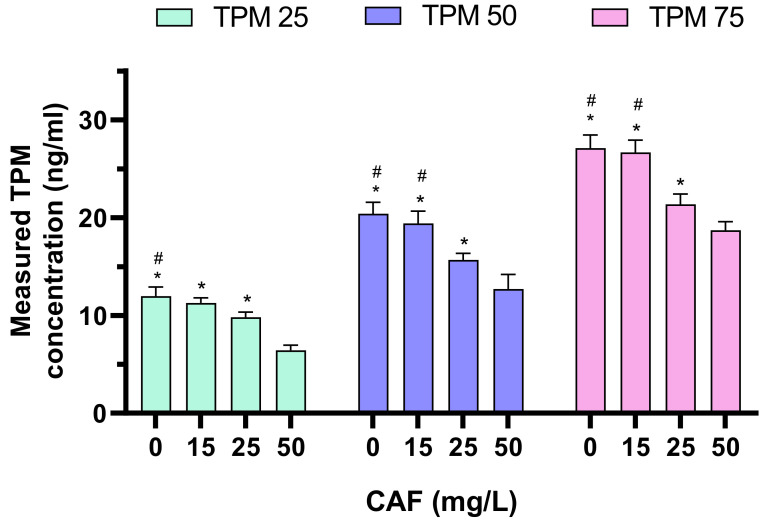
Contents of TPM for the larvae treated with CAF and TPM in a PTZ-induced seizure model; TPM concentrations applied: 25, 50, 75 µm, CAF concentration applied: 15, 25, 50 mg/L. * *p* < 0.05, compared to CAF 50 mg/L group; # *p* < 0.05 compared to CAF 25 mg/L group.

Next, we determined the amount of TPM in larvae treated with both CAF and TPM (Figure 4). Adding 50 mg/L CAF to 25 μM TPM significantly lowered the measured TPM concentration compared to larvae treated with lower CAF doses or solely TPM. Also, adding 25 mg/L CAF reduced the amount of TPM compared to larvae treated solely with 25 μM TPM.

Adding 50 mg/L CAF to 50 μM TPM led to a decrease in the concentrations compared to groups administered 0, 15, or 25 mg/L CAF. Also, treating the animals with 25 mg/L CAF decreased the TPM contents compared to the 0 and 15 mg/L CAF groups during treatment with 50 μM TPM.

Again, in the 75 μM TPM group, 50 mg/L CAF significantly lowered TPM levels compared to lower CAF doses. Also, treating the animals with 25 mg/L CAF decreased the TPM contents compared to the 0 and 15 mg/L CAF groups during treatment with 75 μM TPM.

## 3. Discussion

We investigated the effect of CAF on the anticonvulsant action of TPM in zebrafish larvae in a PTZ-induced seizure model in order to study the interactions of CAF and TPM. We determined whether CAF influences TPM concentrations in a wide range of investigated doses using an analytical method based on ultrahigh-performance liquid chromatography followed by mass spectrometric detection.

### 3.1. The Influence of Caffeine and Topiramate on Larvae Locomotor Activity

Epilepsy stands out as a prevalent and significant neurological disorder, impacting a global population of more than 70 million individuals [15]. In response to the imperative of developing novel therapeutics, diverse animal and genetic epilepsy models have been described based on the purpose of the study [2]. PTZ-induced seizure is the best-described chemical model in both zebrafish and rodents. The effect CAF has on TPM in a chemically induced seizure has already been investigated in some animals, though not in zebrafish [14]. The PTZ model was introduced to the scientific stage in *Danio rerio* in 2005 [13]. It was subsequently confirmed in many studies, including those on antiepileptic drugs [16]. Since then, the zebrafish model has gained interest as a valuable tool in the field of different central nervous system diseases. Afrikanova et al. [16] presented the anticonvulsant activity of TPM against PTZ-induced seizures (20 mM), leading us to choose this model in our study.

In a comprehensive investigation led by Zhang et al., the impact of TPM on zebrafish larvae at different developmental stages was meticulously examined. At 4 dpf, exposure to a 200 μM dose of TPM resulted in a marginal increase in total movement, while a 100 μM dose exhibited no significant effect. Intriguingly, after a 24 h incubation period, no discernible differences were observed compared to the control [17]. Subsequent experimentation involving 6 dpf larvae revealed that a 24 h incubation with TPM at doses of 1, 3, and 10 mM failed to induce significant alterations in total distance in the non-PTZ group. However, a notable decrease in distance was observed in the PTZ model across all three doses [18].

Examining the anticonvulsant properties of TPM, 7 dpf larvae exhibited suppressed seizure-like behavior following an 18 h incubation with a 200 μM dose. Intriguingly, this suppression did not extend to the total seizure duration in the electroencephalogram assay [16] Further insights from a separate study indicated that both prolonged incubation and acute exposure to TPM (200 μM) resulted in conspicuous reductions in locomotor activity in 7 dpf zebrafish [19]. In the PTZ-induced seizure model (10 mM), the total movement was significantly diminished in both TPM-pretreated and acutely exposed groups compared to the controls. The use of a lower PTZ dose (10 mM) over an extended period was a deliberate choice to mitigate the risk of synaptic fatigue, exhaustion, and mortality associated with higher PTZ concentrations, which can lead to a decline in swimming behavior at later time increments [19].

Contrary to the findings of Afrikanova et al., who reported inconsistencies in locomotor behavior and electroencephalogram results [16], the study by Zhang et al. revealed a harmonious reduction in both behavioral and neural activity, meticulously analyzed through electroencephalogram and GCaMP studies [19]. These nuanced observations contribute valuable insights into the multifaceted effects of TPM on zebrafish larvae, emphasizing the significance of considering developmental stages and exposure durations in elucidating the compound’s impact.

Our findings were that a 75 μM dose of TPM significantly defended larvae against PTZ, but did not reduce activity to the levels of the control, which lines up with results in 7 dpf larvae (180 μM, 18 h exposure) [16]. In 7 dpf larvae, 24 h TPM exposure (200 μM) reduced the activity to the control level [19].

There exist a very limited number of studies concerning the interactions between CAF and TPM. Administered acutely or chronically at doses of 23.1 mg/kg and 46.2 mg/kg, CAF increased the amount of TPM, critical to protecting 50% of mice (ED50) from a maximal electroshock (MES)-induced seizure model [20]. Contrarily, lower doses of CAF (5.7 and 11.5 mg/kg) were not found to have an effect on the anticonvulsant action of TPM. Potential pharmacokinetic interactions between the drugs tested were excluded by the authors because, as measured, the free plasma concentration of TPM was not influenced by CAF. It is worth noting that the authors only measured the concentrations of one of the tested compounds (46.2 mg/kg) [20]. CAF at doses of 23.1 mg/kg and 46.2 mg/kg, whether administered acutely or chronically, significantly increased the ED50 value for TPM in rats in MES-induced convulsions [14].

In our study, low doses of CAF did not have an effect on seizures either. Contrary to the abovementioned studies, larger doses of CAF did not cause an increase in the levels of TPM needed to defend the larvae from PTZ, as previously seen in both mice [20] and rats [14]. We found that CAF co-administration decreased movement more than TPM administered alone. Contrary to the abovementioned study in mice, we found that CAF, especially in higher CAF and TPM doses, decreases TPM concentrations. It was previously shown that TPM is the most sensitive antiepileptic drug to CAF; however, any pharmacological interactions have been denied [14]. Assuming no pharmacokinetic interaction, it was proposed that CAF may act as an antagonist to the anticonvulsant properties or that CAF increases seizure susceptibility [14]. The findings provide a foundation for future research and clinical investigations into the therapeutic potential of potential explanation of CAF and TPM interactions.

### 3.2. Caffeine and Topiramate Metabolism

Based on previous research, the formation of the hepatic primordium commences at 28 hpf in zebrafish. This is followed by the expansion of the hepatic structure between 60 and 72 hpf. By the time 120 hpf is reached, liver function, including CYP metabolism, is almost fully established [21]. However, it has been shown that CYP metabolic functions are similar to those of human CYP isoforms even prior to full liver development, confirmed by both mRNA and metabolite assessments in 24–120 hpf larvae [22].

TPM undergoes minimal metabolism and is principally excreted unaltered in urine, amounting to approximately 70% of the administered dose. Six metabolites have been identified in humans, with each contributing less than 5% of the administered dose. It is important to highlight that these TPM metabolites are not known to have any substantial activity [23]. TPM is principally metabolized by enzymes like CYP3A4 and CYP2C19 in the liver, but it is also a weak inhibitor of several other CYP enzymes, such as CYP2C9 and CYP2C19. CAF is metabolized by CYP1A2, and to date, there is no evidence that CAF influences any cytochromes included in TPM metabolism [23].

In summary, comprehending the pharmacokinetic interactions between CAF and TPM is crucial for anticipating how individuals will respond to TPM when they are also using CAF. Additionally, it is essential for pinpointing factors that may impact TPM metabolism. These insights can ultimately guide the development of strategies to ensure the safe and effective use of products containing CAF in patients with epilepsy.

### 3.3. Translating Animal Studies to Humans

While using larvae in experiments offers certain advantages, it requires an understanding of how the feature being studied is affected by the development of the animal. When using paracetamol, it was observed that absorption increased by 106% between 3 and 4 dpf, but did not increase significantly at 5 dpf [24]. Conversely, the elimination of drugs grew by 17.5% per day within 3 to 5 dpf due to the ongoing development of enzymatic processes and eliminating organs. *Danio rerio* larvae have lower metabolic rates than poikilotherms, which may lead to variations in clearance values. However, the correlation in higher vertebrates strengthens with age, with the best fit observed at 5 dpf [24].

As the majority of experiments in pharmacology and toxicology are conducted during these initial phases, it becomes imperative to grasp and assess the influence of development, including the extent to which a single day of the experiment can alter the internal exposure to exogenous substances. One potential approach to address this is by determining the internal concentration of the tested compound in zebrafish larvae, a method we have used in this study.

### 3.4. Future Perspectives

Pharmacological intervention achieves seizure control in approximately 80% of individuals diagnosed with epilepsy. The factors contributing to suboptimal response to antiepileptic drugs in some patients remain largely unidentified [25]. There are several case reports that suggest that CAF can trigger seizures in individuals with epilepsy. The newest one from Bonilha and Li is a case report of a man whose daily habit of heavy drinking was associated with an increased seizure frequency. The intervention in the form of stopping caffeine consumption resulted in a significant decrease in the frequency of seizures [26]. Nevertheless, this constitutes a singular case report. As previously mentioned, there are limited clinical data regarding the association between CAF and epileptic seizures. As of yet, no studies related to the key term “epilepsy and caffeine” have been registered on the international clinical research database ClinicalTrials.gov [27]. There have been a few clinical trials on the effect of caffeine on seizures. Of note is an extensive questionnaire-based study whose aim was to examine the association of cigarette smoking, caffeine use, and alcohol intake with risk of seizure or epilepsy among women aged 25–42 years. The study was conducted in over 100,000 nurses. Individuals who reported seizures or epilepsy did not exhibit a distinct caffeine intake compared to the overall cohort [28]. Compared to animal studies, the clinical trials mentioned earlier had some limitations. The methodology has constraints, as variations in the size of cups and caffeine concentration likely differed among studies and participants. Additionally, reliance on self-reports in these studies introduces sensitivity to recall and response biases. Studies that exclusively enrolled women may also be susceptible to selection bias [28,29].

In conclusion, clinical studies on the impact of caffeine consumption on the efficacy of antiepileptic drugs are limited and come with inherent constraints. Although the *Danio rerio* model caters to the needs of scientific groups due to its accessibility, ease of reproduction, cost-effectiveness, and potential for translatability to the human model, it also has limitations, as outlined in this paper. There is an urgent need for well-designed clinical studies that can address the question posed by numerous research groups worldwide: “Does the dose and quantity of caffeine influence the incidence of seizures?”.

## 4. Materials and Methods

### 4.1. Animals

*Danio rerio* stocks of a wild-type zebrafish strain (AB strain, Experimental Medicine Centre, Medical University of Lublin, Poland) were maintained at a temperature of 26–28.5 °C in a controlled environment (pH ranging between 6.9 and 7.5; conductivity of 550–700; 14/10 h light/dark cycle). Embryos were reared under a standard light/day cycle in an E3 embryo medium in an incubator (IN 110 Memmert GmbH, Buechenbach, Germany). The 4-day post-fertilization (dpf) zebrafish larvae were used for the assays. After the experiment, larvae were immediately killed by immersion in a solution of tricaine (15 μM). All experiments were conducted following the National Institute of Health Guidelines for the Care and Use of Laboratory Animals and the European Community Council Directive for the Care and Use of Laboratory Animals of 22 September 2010 (2010/63/EU). For experiments on larvae up to 5 dpf, agreement with the local ethics commission is not required.

### 4.2. Chemicals

TPM, CAF, and PTZ were purchased from Sigma-Aldrich (Saint Louis, MO, USA). All compounds were dissolved in deionized water and diluted in E3 embryo medium (pH 7.1–7.3; 17.4 μM NaCl, 0.21 μM KCl, 0.12 μM MgSO_4_ and 0.18 μM Ca(NO_3_)_2_) to achieve a designated concentration.

### 4.3. Evaluation of Locomotor Behavior

Larvae were preincubated in 100 mL of E3 embryo medium or tested substances for 18 h in individual wells of a 96-well plate at 28 °C. Ten larvae were used per treatment parameter and per experiment. After the preincubation, 100 mL of E3 embryo medium or 100 mL of a 40 mM PTZ solution was added to obtain a final concentration of 20 mM to evoke seizures [16]. Larvae were allowed to habituate for 5 min in a dark chamber of an automated tracking device (ZebraBox^TM^ apparatus; Viewpoint, Lyon, France). The total locomotor activity was then quantified using ZebraLab^TM^ software (https://www.viewpoint.fr/product/zebrafish/fish-behavior-monitoring/zebralab, accessed on: 20 October 2023, Viewpoint, Lyon, France) [16]. Average movement or activity was expressed in “actinteg” units. The actinteg value of the ZebraLab^TM^ software is defined as the sum of all image pixel changes detected during the time slice defined for the experiment (30 min). All tracking experiments were performed in triplicate.

### 4.4. Treatments for the Assessment of Caffeine and Topiramate Concentrations

Larvae were preincubated in 500 μL of E3 embryo medium or tested substances for 18 h in a 48-well plate (5 larvae per plate) at 28 °C. After the preincubation, 250 μL of E3 embryo medium or 250 μL of a 60 mM PTZ solution was added to obtain a final concentration of 20 mM to evoke seizures. Larvae were allowed to incubate for 30 min with PTZ.

### 4.5. Sample Preparation

Larvae were washed with E3 embryo medium three times, placed in a tube (20 larvae per sample) and homogenized by sonication (4 × 5 s) in 150 μL 100 mM NH_4_HCO_3_. Then, samples were incubated on ice for 15 min and centrifuged (15,000× *g*, 15 min, 4 °C), after which 100 μL of supernatant was mixed with a methanol:ethanol solution (1:1) to obtain a 1:3 ratio, respectively, and vortexed for 30 s. After 15 min incubation at 20 °C and centrifugation (15,000× *g*, 10 min, 4 °C), 250 μL of supernatant was placed in a chromatography vial. Eight-level standard solutions spiked into control larvae homogenate were used to construct the calibration curves.

### 4.6. LC-QQQ-MS Analysis

Chromatographic separation was conducted using an Agilent 1290 Infinity II LC system coupled to an Agilent 6470 Triple Quad tandem mass spectrometer equipped with electrospray ionization source Jet Stream Technology (Agilent Technologies, Santa Clara, CA, USA). Analyte determination was performed using dynamic multiple reaction monitoring (dMRM) in positive ionization mode. The chromatographic separations were conducted using an RRHD Zorbax Eclipse Plus C18 column (Agilent Technologies; 2.1 mm × 50 mm; 1.8 μm) at a flow rate of 0.3 mL/min. The mobile phases were 0.1% formic acid in water (A) and 0.1% formic acid in methanol (B). The run time was 7 min, with a gradient program of 0–4 min, 13–18% B; 4.01–6 min, 60–75% B; 6.01–7 min, 95% B, column conditioning 3 min. The injection volume was 1 μL and the column temperature was maintained at 40 °C. The mass spectrometer was operated in positive electrospray ionization mode (ESI+) with the following settings: ion source gas, N_2_; ion source gas temperature, 300 °C; ion source gas flow rate, 12 L/min; nebulizer pressure, 40 psi; sheath gas temperature, 350 °C; sheath gas flow rate, 12 L/min; and capillary voltage, 4000 V. After each injection, the sampling needle was washed with a mixture of methanol and water (80:20, *v*:*v*). The MS parameters, such as product ion, fragmentor and collision energy, were optimized and are provided in Table 1. The data were acquired using Mass Hunter Acquisition B 09.00 software and processed using Mass Hunter Quantitative 10.02 software (Agilent Technologies, Santa Clara, CA, USA).

### 4.7. Statistical Analysis

Statistical analyses were performed in GraphPad Prism 8 (GraphPad Software, San Diego, CA, USA). For comparison, analysis of variance was performed (one-way or two-way ANOVA). One-way ANOVA was followed by Tukey’s test (post hoc test). In the case of two-way ANOVA, Bonferroni’s test was used as a post hoc test. The confidence limit of *p* < 0.05 was considered statistically significant. Data are presented as means ± standard deviation (SD). Zebrafish larvae were randomly allocated to experimental groups. The experiments were performed in triplicate.

### 4.8. Study Limitations

The studies discussed previously show contradictory results, which may be a result of the use of different models referring to different epilepsy types, various methods for inducing seizures, differing methods of administering drugs, varying doses of chemical substances, and different measurement devices, acquirement methods, and parameters assessed.

## 5. Conclusions

This study aimed to investigate the influence of CAF and TPM combinations on larval locomotor activity in a PTZ-induced seizure model using zebrafish larvae. We examined the effects of different concentrations of TPM and CAF, both individually and in combination, on larval movement. Additionally, we quantified the levels of TPM in zebrafish larvae treated with different doses of TPM alone or in combination with CAF.

- CAF, when administered in combination with TPM, exerted a nuanced influence on larval locomotor activity.
- Higher concentrations of CAF (50 mg/L) demonstrated a suppressive effect on zebrafish activity across all investigated TPM doses, with maximal protection achieved in conjunction with 75 μM TPM.
- CAF, especially with higher CAF and TPM doses, decreases the TPM concentration in the PTZ-induced seizure model in zebrafish.

This study highlights the importance of comprehending drug metabolism and considering developmental aspects when translating findings from zebrafish larvae to human contexts. While the study adds valuable insights to the understanding of CAF and TPM interactions, it underscores the necessity for well-designed clinical studies to elucidate the impact of CAF consumption on antiepileptic drug efficacy in humans. Our results provide a potentially new explanation for the effect of CAF on TPM that has not been previously considered. As previously proposed, unless proven otherwise, CAF consumption should be considered a potential factor that could aid in controlling epileptic seizures [14].

## Figures and Tables

**Table 1:** MRM parameters for topiramate.

Analyte	Formula	Retention Time (min)	Retention Window (min)	Precursor Ion *m*/*z*	Product Ion *m*/*z*	Fragmentor (V)	Collision Energy (V)
Topiramate	C_12_H_21_NO_8_S	5.83	0.5	340.1	184	136	13
				340.1	264	136	5

## Data Availability

The data presented in this study are available in the manuscript.
